# Trends in LDL-C and Non-HDL-C Levels with Age

**DOI:** 10.14336/AD.2019.1025

**Published:** 2020-10-01

**Authors:** Peng Zhang, Qian Su, Xiaomiao Ye, Ping Guan, Chengjun Chen, Yanwen Hang, Jian Dong, Zhongjie Xu, Wei Hu

**Affiliations:** ^1^Department of Cardiology, Minhang Hospital, Fudan University, Shanghai, China; ^2^Shanghai Minhang District Medical Emergency Center, Shanghai, China

**Keywords:** low-density lipoprotein cholesterol (LDL-C), non-high-density lipoprotein cholesterol (non-HDL-C), age

## Abstract

Understanding how blood lipid levels change with age in the general population is a precondition to defining dyslipidemia. To explore age-related trends in LDL-C and non-HDL-C levels in the general population, a large-scale cross-sectional study with 49,201 males and 35,084 females was adopted. Trends of non-HDL-C and LDL-C levels were plotted against each age (18 to 85 years old, one-year increments); the trends, as well as the influence of confounding factors on the trends, were validated and adjusted by linear regression modeling. The trajectory of LDL-C and non-HDL-C levels by age displayed a nonlinear correlation trend. Further multivariate linear regression modeling that incorporated sex-specific age phases showed that age was positively associated with LDL-C and non-HDL-C levels, with coefficients of 0.018 and 0.031, respectively, in females aged ≥18 to ≤56 years and negatively associated with LDL-C and non-HDL-C levels, with coefficients of -0·013 and -0.015, respectively, in females aged ≥57 years. The LDL-C and non-HDL-C levels increased with age in males ≥18 to ≤33 years of age, with coefficients of 0.025 and 0.053, respectively; the lipid levels plateaued at ≥34 to ≤56 years of age and subsequently decreased in those ≥57 years of age, with coefficients of -0.008 and -0.018, respectively. In contrast, pooled analyses without age stratification concealed these details. In conclusion, fluctuating increasing and decreasing lipid levels occurred with phases of aging in both sexes. Well-grounded age stratification is necessary to improve lipid-related pathophysiological studies.

Dyslipidemia, defined as an unfavorable blood-lipid profile, plays an important role in the development and progression of coronary heart disease (CHD) [1]. Epidemiological studies have shown that high levels of total cholesterol, low-density lipoprotein cholesterol (LDL-C) and non-high-density lipoprotein cholesterol (non-HDL-C) are important lipid-related risk factors for CHD [1-6]. The National Lipid Association Expert Panel concluded that elevated levels of non-HDL-C and LDL-C are a root cause of atherosclerosis because the key process underlying their elevation contributes to most clinical CHD events [1,7].

Historically, consensus regarding the role of elevated LDL-C levels in CHD development and progression has been gradually shaped since the 1980s [5]. Although multicenter, randomized and controlled trials are believed to provide the highest level of evidence to understand causality between high LDL-C levels and CHD, the conclusions of such studies have not been fully approved in practice. Existing problems are mainly reflected in 1) the absence of the LDL-C level from most CHD risk-estimation equations is puzzling [8-10]; and 2) current canonical treatments, a combination of lifestyle intervention and pharmacotherapy, aimed at lowering plasma LDL-C level, reducing blood pressure, and preventing thrombotic incidence, fails to normalize risk in individuals at high risk of CHD [11]. This evidence implies that other CHD risk factors beyond LDL-C or non-HDL-C, such as age and sex, may deserve attention. In addition, the performance of CHD risk estimators and the effect of statin therapy might be greatly weakened if we are ignorant to the age and sex distribution characteristics of known risk factors. The current optimal (LDL-C < 3.36 mmol/L; non-HDL-C < 3.3 mmol/L), borderline (3.36 ≤ LDL-C ≤ 4.11 mmol/L; 3.3 ≤ non-HDL-C ≤ 4.1 mmol/L), and high (LDL-C > 4.11 mmol/L; non-HDL-C > 4.11 mmol/L) LDL-C and non-HDL-C levels recommended by the National Lipid Association were decided without adequately considering sex differences or the changes in LDL-C and non-HDL-C levels with age [1]. The National Health and Nutrition Examination Survey data showed that the trends in lipid levels among adults in the US have been generally beneficial [12-15], but these studies did not address the role of aging.

Aging is an inherent and fundamental physiological process characterized by deterioration in every facet of a biological system over time [16,17]. The absorption, synthesis, and metabolism of fat and lipoprotein also change with age via complex mechanisms [17]. An understanding of the impact of aging on blood-lipid levels in the general population is a precondition to defining dyslipidemia and determining the role of lipids in CHD. Unfortunately, few studies have focused on age-related blood lipid trends in the general population to date.

To determine the trends in lipid levels with age, a large-scale cross-sectional study with 49,201 males and 35,084 females was adopted. The trajectories of LDL-C and non-HDL-C levels were plotted with age ranging from 18 to 85 years old; the trends and any influence of confounding factors on the trends were validated and adjusted by fitted linear regression modeling. Our study will improve the current definition, prevention and therapy of dyslipidemia.

## MATERIALS AND METHODS

### Ethics

This study was conducted in accordance with the statement of ethical principles for medical research involving human subjects by the World Medical Association Declaration of Helsinki and was approved by the Internal Review Board of Minhang Hospital, Fudan University, Shanghai, China. Written informed consent was obtained according to the guidelines of the National Ethics Regulation Committee.

### Study design and participants

**Figure 1.**
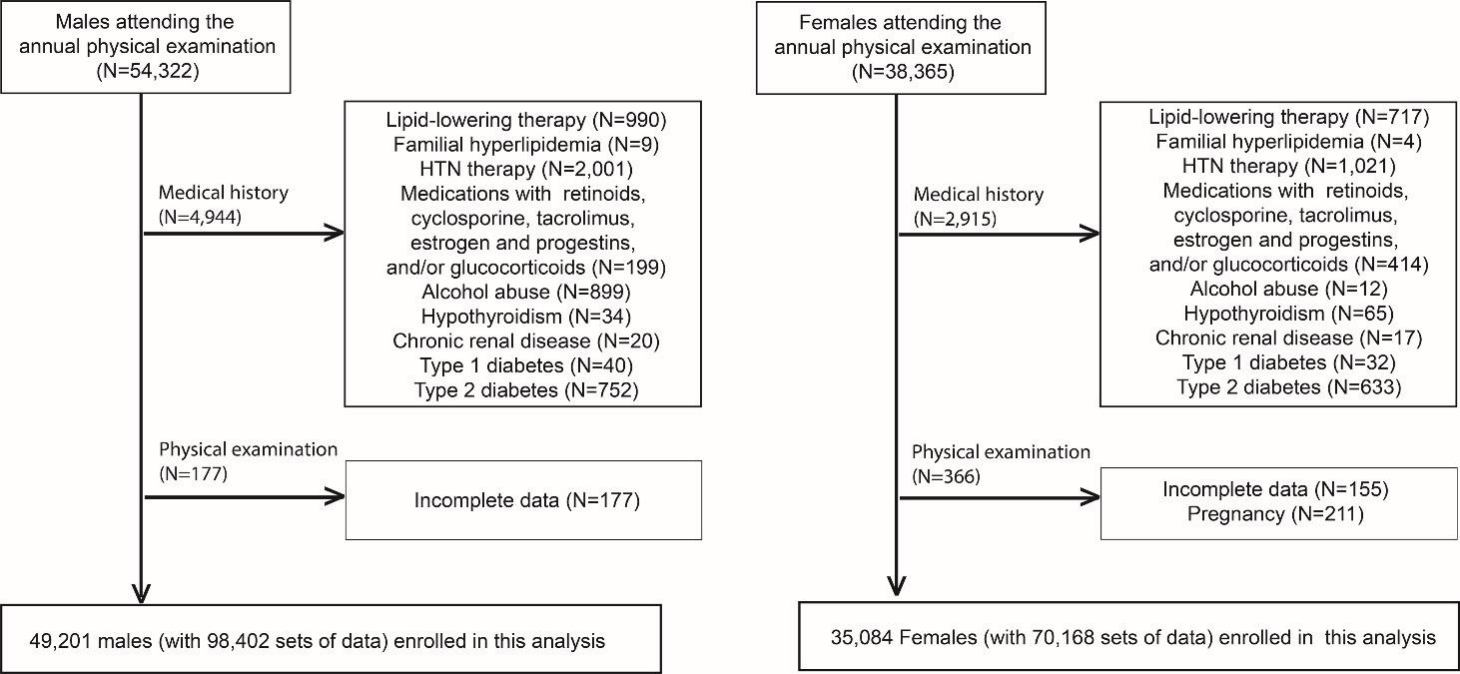
Flow chart of the participants. A total of 92,687 subjects (54,322 males and 38,365 females) attended annual physical examinations, of whom 84,285 subjects (49,201 males and 35,084 females) with 168,570 person times data were included in the statistical analyses.

This cross-sectional study involved seemingly healthy subjects from the general population with stratification by sex and age and adjustment for known confounding factors. Subjects aged between 18 to 85 years who underwent a physical examination in our hospital in 2016 and 2017 were enrolled. In total, 54,322 males and 38,365 females initially attended annual physical examinations. Subjects who were undergoing lipid-lowering or antihypertensive therapy or who were taking retinoids, cyclosporine, tacrolimus, and/or glucocorticoids were excluded, as were those with known familial hyperlipidemia, cardiovascular disease, hypothyroidism, chronic renal disease, or type 1 or type 2 diabetes. Subjects who were alcohol abusers and pregnant women were also excluded. Finally, 49,201 males and 35,084 females were involved in this study (Fig. 1). Use of the subjects’ biannual checkup data resulted in a collection of 168,570 person times of data (2×49,201 + 2×35,084). Some of the subjects were missing one or more parameters; therefore, the sample sizes used in the analyses described below differ slightly.

### Anthropometric measurements, questionnaires, and definitions

Body mass index (BMI) was calculated as weight divided by height in meters squared (kg/m^2^). Smoking status, level of alcohol consumption, and lifestyle factors were recorded using a questionnaire. Smoking was defined as smoking one or more cigarettes daily for at least 1 year. According to the 2015-2020 Dietary Guidelines for Americans of the US Departments of Health and Human Services and of Agriculture, moderate alcohol consumption is defined as up to one alcoholic drink per day for females and up to two alcoholic drinks per day for males [18]. Physical activity was assessed using the Baecke Questionnaire for Habitual Physical Activity [19]. Waking blood pressure was measured in the seated position after a 5 min rest. The mean of three measurements taken at 1 min intervals was recorded [20].

### Laboratory biochemical parameters

After fasting for at least 8 h, a fasting blood sample was drawn from the antecubital vein in the morning. Cholesterol concentrations were directly measured using the VerticalAuto Profile (Atherotech, London, UK), an inverted rate-zonal, single-vertical spin, density-gradient ultracentrifugation technique that separates lipoprotein subfractions and measures the levels of LDL-C, very low-density lipoprotein cholesterol (VLDL-C), and high-density lipoprotein cholesterol (HDL-C). Non-HDL-C, calculated as total-C - HDL-C, is the total quantity of cholesterol carried by all potentially atherogenic, apo B-containing lipoprotein particles, including LDL, IDL, Lp(a), VLDL (including VLDL remnants), chylomicron particles and remnants. The serum glucose level was measured using an H-7600 autoanalyzer (Hitachi, Tokyo, Japan).

### Statistical analysis

All variables, except TG, generally fit a normal distribution as assessed by frequency distribution histograms. Normally distributed data were presented as the means ± standard deviation (SD), skewed data were presented as the median (interquartile range), and categorical data were presented as the count (percentage). The significance of differences between groups was examined by *t*-tests, one-way analysis of variance, or χ2 tests according to the distribution of the data.

We used LDL-C and non-HDL-C to assess the trends of blood-lipid levels for the following reasons: there was multicollinearity between blood lipids due to their biochemical similarity, and both are classified as atherogenic cholesterols [1]. The levels of LDL-C and non-HDL-C were individually used as the dependent variable in separate linear regression analyses.

The mean LDL-C and non-HDL-C levels were calculated and plotted by each age. The turning point of the lipid trajectories was determined by curve trend observation and Duncan analyses of the group means for homogeneous subsets. The significance of the age stratification was validated using linear regression modeling.

The associations between lipids and age were quantified by performing linear regression analyses stratified by sex and age. To assess the influence of confounding factors—such as BMI, blood pressure (BP), glucose level, lifestyle, moderate drinking and smoking—on the correlation between blood-lipid levels and age, the associations between these parameters and LDL-C/non-HDL-C levels were first evaluated by univariable linear regression; potential independent variables according to the univariable analysis (p<0.10) were introduced into each starting model during the multivariate linear regression modeling; variables were then eliminated manually using the backward step-by-step approach, depending on the largest *P* value; any factor remaining in the final equations following the multivariate linear regression modeling was considered to be independently associated with the LDL-C and/or non-HDL-C level.

We considered P-values < 0.05 by a two-sided test to be indicative of statistical significance. Statistical analyses were performed using SPSS ver. 17.0.0 (SPSS, Chicago, IL, USA).

**Table 1:** General distribution characteristics of the parameters by sex.

Parameter	Male	Female	*P*
Number of subjects	49,201	35,084	*/*
Person-time	98,402	70,168	*/*
Age, years	44.3 ± 13.2	42.4 ± 12.5	<*0.001*
Current smoker	17,221 (35.0%)	726 (2.1%)	<*0.001*
Moderate drinker	10,993 (22.3%)	1,022 (2.9%)	<*0.001*
Inactive lifestyle	8,856 (18.0%)	5,101 (14.8%)	<*0.001*
BMI (kg/m^2^)	24.4 ± 3.1	22.3 ± 3.1	<*0.001*
Systolic BP	132.4 ± 17.7	125.0 ± 19.0	<*0.001*
Diastolic BP	81.8 ± 11.3	75.1 ± 11.2	<*0.001*
TC (mmol/L)	4.76 ± 0.91	4.59 ± 0.87	<*0.001*
TG (mmol/L)	1.73 (1.20, 2.56)	1.17 (0.84, 1.68)	<*0.001*
HDL-C (mmol/L)	1.29 ± 0.38	1.49 ± 0.32	<*0.001*
LDL-C (mmol/L)	2.59 ± 0.82	2.46 ± 0.73	<*0.001*
Non-HDL-C (mmol/L)	3.48 ± 0.96	3.10 ± 0.85	<*0.001*
Glucose (mmol/L)	5.52 ± 1.4	5.23 ± 1.0	<*0.001*

Normally distributed data are presented as the means ± standard deviation (SD), skewed data are presented as the median (interquartile range), and categorical data are presented as the count (percentage). Abbreviations: BMI, Body mass index; TC, total cholesterol; TG, triglycerides; HDL-C, high density lipoprotein cholesterol; LDL-C, low density lipoprotein cholesterol; BP, blood pressure.

## RESULTS

### Participants

In total, 54,322 males and 38,365 females attended an annual physical examination for 2 consecutive years; 4,944 males and 2,915 females were excluded by medical history; 177 males and 366 females were further excluded due to incomplete data or pregnancy. Finally, 49,201 males and 35,084 females (168,570 sets of data) were analyzed (Figure 1A). Within the pooled cohort of males and females, the average age, BMI, systolic BP, diastolic BP, LDL-C levels, non-HDL-C levels, and glucose levels were significantly higher in males than in females, and males had higher rates of smoking, moderate alcohol consumption, and an inactive lifestyle (Table 1).

### Age-related trends of LDL-C and non-HDL-C levels

**Figure 2.**
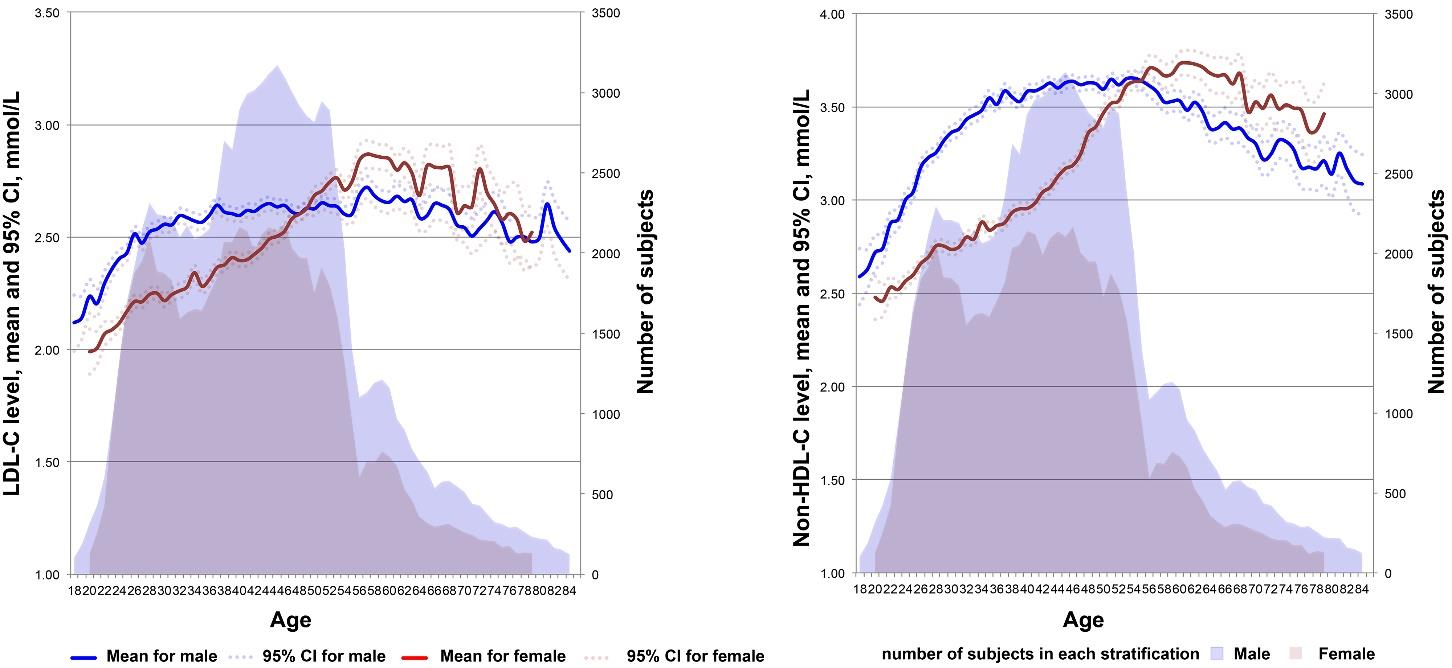
Lifetime trends of the LDL-C and non-HDL-C levels. Means with 95% CIs for LDL-C and non-HDL-C levels (left ordinate) are plotted against each age by sex; the shadow indicates the number of subjects at each age (right ordinate).

To learn the dynamic lifespan trends of LDL-C and non-HDL-C levels, the mean levels were plotted against age. As shown in Figure 2, fluctuating curve trends of lipid level with age were observed. Among males, the LDL-C and non-HDL-C levels increased from 2.12 ± 0.64 and 2.59 ± 0.77 mmol/L at 18 years of age to 2.59 ± 0.80 and 3.46 ± 0.93 mmol/L, respectively, at 33 years of age. This was followed by a plateau phase until 56 years of age with LDL-C maintaining at 2.57-2.69 mmol/L and non-HDL-C maintaining at 3.48-3.64 mmol/L and then a decrease to 2.44 ± 0.75 and 3.09 ± 0.88 mmol/L, respectively, at 85 years of age. Among females, the LDL-C and non-HDL-C levels increased gradually from 2.07 ± 0.50 and 2.50 ± 0.60 mmol/L, respectively, at 18 years of age to 2.84 ± 0.81 and 3.71 ± 0.86 mmol/L, respectively, at 56 years of age and subsequently entered a volatility phase of overall decline and decreased to 2.45 ± 0.76 and 3.15 ± 0.79 mmol/L, respectively, at 85 years of age.

In general, although the average LDL-C and non-HDL-C levels did not exceed the high borderline levels (3.36 and 4.10 mmol/L, respectively) in each age, the means+SDs of LDL-C and non-HDL-C levels exceeded the high borderline levels approximately 10 and 20 years earlier, respectively, in males than in females (39 *vs*. 49 and 28 *vs*. 48 years, respectively).

After integrating the dynamic trajectories of LDL-C and non-HDL-C levels and the Duncan analyses on means, the changes in LDL-C and non-HDL-C levels with age were divided into increasing (≥ 18 to ≤ 33 years), plateau/slow increasing (≥ 34 to ≤ 56 years), and decreasing (≥ 57 years) phases among males and increasing (≥ 18 to ≤ 56 years) and decreasing (≥ 57 years) phases among females in the subsequent validation analyses.

### Linear analyses validated the significance and necessity of age stratification

The coefficients of age in association with LDL-C and non-HDL-C levels were initially quantified by univariate linear regression analysis in pooled and age-stratified populations without adjustments with confounding factors.

Among females, the univariate linear regression coefficients of the total population, the ≥ 18 to ≤ 56-year age group, and ≥ 57-year age group for LDL-C were 0.016, 0.020, and -0.013, respectively. Therefore, the LDL-C level increased with age in females < 57 years of age and decreased with age in those ≥ 57 years of age. Among males, the coefficients of the total population, the ≥ 18 to ≤ 33-year age group, the ≥ 34 to ≤ 56 age group, and ≥ 57-year age group for LDL-C were 0.003, 0.026, 0.002, and -0.008, respectively. Thus, the LDL-C level of males increased with age from 18 to 33 years of age, plateaued at 34 to 56 years of age, and decreased at ≥ 57 years of age (Table 2).

The non-HDL-C level increased with age in females < 57 years of age and decreased with age in those ≥ 57 years of age. The linear regression coefficients of the total population, the ≥ 18 to ≤ 56-year age group, and the ≥ 57-year age group were 0.026, 0.033, and -0.014, respectively. Among males, the coefficients of the total population, the ≥ 18 to ≤ 33-year age group, the ≥ 34 to ≤ 56-year age group, and the ≥ 57-year age group were 0.005, 0.052, 0.005, and -0.017, respectively. Thus, the non-HDL-C level increased with age in males ≥ 18 to ≤ 33 years of age, plateaued at 34 to 56 years of age, and decreased with age at ≥ 57 years of age (Table 3).

In summary, the univariate linear regression analyses validated the significance and necessity of the age ranges we established in the associations between the non-HDL-C/LDL-C levels and age in both sexes. Pooled analyses without age stratification concealed the detailed age-range-specific correlations between LDL-C/non-HDL-C levels and age.

### Associations between confounding factors and LDL-C/non-HDL-C levels evaluated by univariate linear regression analyses

The associations of blood pressure (BP), glucose level, BMI, lifestyle, drinking and smoking with LDL-C and non-HDL-C levels were also evaluated by univariate linear regression analysis in pooled and age-stratified populations.

Among females, systolic BP, moderate drinking and smoking were not associated with LDL-C level or non-HDL-C level (Table 2 and Table 3). Diastolic BP, glucose, BMI and an inactive lifestyle displayed significant associations with LDL-C and non-HDL-C levels (Table 2 and Table 3). Among males, glucose, BMI, inactive lifestyle, moderate drinking and smoking displayed significant associations with LDL-C and non-HDL-C levels (Table 2 and Table 3). Systolic BP was not associated with LDL-C levels, but it was associated with non-HDL-C levels in males aged 18 to 33 years old (Table 2 and Table 3). Diastolic BP was associated with LDL-C and non-HDL-C levels in males ≥ 34 years old (Table 2 and Table 3).

**Table 2:** Associations with LDL-C level evaluated by univariate linear regression analyses.

Female					Male			
Variable	Coef.	95% CI	*P*		Variable	Coef.	95% CI	*P*
*Pooled*								
Age	0.016	0.015~0.016	<*0.001*		Age	0.003	0.002~0.003	<*0.001*
Systolic BP	/	/	*/*		Systolic BP	/	/	*/*
Diastolic BP	0.007	0.003~0.012	<*0.001*		Diastolic BP	0.005	0.002~0.009	<*0.001*
Glucose (mmol/L)	0.091	0.023~0.132	<*0.001*		Glucose (mmol/L)	0.082	0.045~0.124	*0.003*
BMI (kg/m^2^)	0.031	0.022~0.044	*0.001*		BMI (kg/m^2^)	0.041	0.032~0.056	<*0.001*
Inactive lifestyle	0.023	0.019~0.038	*0.002*		Inactive lifestyle	0.031	0.019~0.044	*0.003*
Moderate drinking	/	/	*/*		Moderate drinking	0.044	0.032~0.056	*0.003*
Smoking	/	/	*/*		Smoking	0.056	0.045~0.077	*0.001*
*Age-stratified*								
*Age phase I: 18 ≤ age ≤ 56 (N = 61,420)*					*Age phase I: 18 ≤ age ≤ 33 (N = 22,942)*			
Age	0.020	0.020~0.021	<*0.001*		Age	0.026	0.023~0.029	<*0.001*
Systolic BP	/	/	*/*		Systolic BP	/	/	*/*
Diastolic BP	0.004	0.001~0.008	<*0.001*		Diastolic BP	/	/	*/*
Glucose (mmol/L)	0.114	0.098~0.143	*0.002*		Glucose (mmol/L)	0.154	0.137~0.176	*0.001*
BMI (kg/m^2^)	0.039	0.028~0.051	*0.003*		BMI (kg/m^2^)	0.063	0.048~0.079	<*0.001*
Inactive lifestyle	0.048	0.039~0.066	<*0.001*		Inactive lifestyle	0.023	0.019~0.033	*0.004*
Moderate drinking	/	/	*/*		Moderate drinking	0.032	0.023~0.054	<*0.001*
Smoking	/	/	*/*		Smoking	0.066	0.056~0.076	<*0.001*
					*Age phase II: 34 ≤ age ≤ 56 (N = 59,807)*
					Age	0.002	0.001~0.003	<*0.001*
					Systolic BP	/	/	*/*
					Diastolic BP	0.008	0.004~0.011	*0.002*
					Glucose (mmol/L)	0.032	0.021~0.039	<*0.001*
					BMI (kg/m^2^)	0.029	0.019~0.045	*0.002*
					Inactive lifestyle	0.016	0.012~0.023	*0.002*
					Moderate drinking	0.056	0.043~0.077	<*0.001*
					Smoking	0.103	0.078~0.201	*0.003*
*Age phase II: age ≥ 57 (N = 8,748)*					*Age phase III: age ≥ 57 (N = 15,653)*			
Age	-0.013	-0.010 ~ -0.007	<*0.001*		Age	-0.008	-0.010 ~ -0.007	<*0.001*
Systolic BP	/	/	*/*		Systolic BP	/	/	*/*
Diastolic BP	0.013	0.009~0.017	<*0.001*		Diastolic BP	0.004	0.001~0.008	*0.027*
Glucose (mmol/L)	0.049	0.032~0.076	<*0.001*		Glucose (mmol/L)	0.029	0.021~0.043	*0.003*
BMI (kg/m^2^)	0.017	0.012~0.032	<*0.001*		BMI (kg/m^2^)	0.019	0.011~0.044	<*0.001*
Inactive lifestyle	0.018	0.012~0.033	*0.003*		Inactive lifestyle	0.023	0.014~0.033	<*0.001*
Moderate drinking	/	/	*/*		Moderate drinking	0.021	0.014~0.034	<*0.001*
Smoking	/	/	*/*		Smoking	0.018	0.016~0.034	*0.003*

Coef., the coefficient indicating a one-unit increase in a variable according to the univariate linear regression analyses; 95% CI, 95% confidence interval. "/" indicates that a variable is not significantly associated with LDL-C level in a given model.

**Table 3:** Associations with non-HDL-C level evaluated by univariate linear regression analyses.

Female					Male			
Variable	Coef.	95% CI	*P*		Variable	Coef.	95% CI	*P*
*Pooled*								
Age	0.026	0.026~0.027	<*0.001*		Age	0.005	0.004~0.005	<*0.001*
Systolic BP	/	/	*/*		Systolic BP	/	/	*/*
Diastolic BP	0.021	0.017~0.033	*0.003*		Diastolic BP	0.005	0.003~0.017	*0.005*
Glucose (mmol/L)	0.015	0.011~0.021	*0.004*		Glucose (mmol/L)	0.211	0.139~0.341	*0.004*
BMI (kg/m^2^)	0.054	0.039~0.061	<*0.001*		BMI (kg/m^2^)	0.079	0.067~0.087	<*0.001*
Inactive lifestyle	0.031	0.022~0.041	*0.003*		Inactive lifestyle	0.054	0.034~0.069	*0.003*
Moderate drinking	/	/	*/*		Moderate drinking	0.049	0.041~0.051	<*0.001*
Smoking	/	/	*/*		Smoking	0.068	0.056~0.099	*0.008*
*Age-stratified*								
*Age phase I: 18 ≤ age ≤ 56 (N = 61,420)*					*Age phase I: 18 ≤ age ≤ 33 (N = 22,942)*			
Age	0.033	0.033~0.034	<*0.001*		Age	0.052	0.049~0.055	<*0.001*
Systolic BP	/	/	*/*		Systolic BP	0.005	0.002~0.023	*0.027*
Diastolic BP	0.029	0.021~0.031	<*0.001*		Diastolic BP	/	/	*/*
Glucose (mmol/L)	0.201	0.178~0.231	<*0.001*		Glucose (mmol/L)	0.312	0.201~0.402	*0.004*
BMI (kg/m^2^)	0.058	0.048~0.068	<*0.001*		BMI (kg/m^2^)	0.079	0.056~0.087	<*0.001*
Inactive lifestyle	0.028	0.021~0.039	<*0.001*		Inactive lifestyle	0.036	0.026~0.057	*0.004*
Moderate drinking	/	/	*/*		Moderate drinking	0.034	0.023~0.045	<*0.001*
Smoking	/	/	*/*		Smoking	0.079	0.065~0.101	*0.008*
					*Age phase II: 34 ≤ age ≤ 56 (N = 59,807)*
					Age	0.005	0.004~0.006	<*0.001*
					Systolic BP	/	/	*/*
					Diastolic BP	0.007	0.005~0.011	*0.006*
					Glucose (mmol/L)	0.192	0.136~0.203	<*0.001*
					BMI (kg/m^2^)	0.061	0.053~0.072	<*0.001*
					Inactive lifestyle	0.101	0.079~0.209	*0.002*
					Moderate drinking	0.063	0.045~0.076	*0.008*
					Smoking	0.114	0.094~0.203	*0.009*
*Age phase II: age ≥ 57 (N = 8,748)*		*Age phase III: age ≥ 57 (N = 15,653)*
Age	-0.014	-0.017~-0.011	<*0.001*		Age	-0.017	-0.019~-0.015	<*0.001*
Systolic BP	/	/	*/*		Systolic BP	/	/	*/*
Diastolic BP	0.009	0.005~0.013	*0.004*		Diastolic BP	0.006	0.003~0.023	*0.034*
Glucose (mmol/L)	0.065	0.054~0.105	*0.003*		Glucose (mmol/L)	0.114	0.098~0.231	*0.006*
BMI (kg/m^2^)	0.041	0.038~0.059	<*0.001*		BMI (kg/m^2^)	0.042	0.037~0.053	<*0.001*
Inactive lifestyle	0.031	0.022~0.044	<*0.001*		Inactive lifestyle	0.041	0.029~0.057	*0.005*
Moderate drinking	/	/	*/*		Moderate drinking	0.041	0.036~0.055	*0.003*
Smoking	/	/	*/*		Smoking	0.031	0.019~0.065	*0.007*

Coef., the coefficient indicating a one-unit increase in a variable according to the univariate linear regression analyses; 95% CI, 95% confidence interval. "/" indicates that a variable is not significantly associated with non-HDL-C level in a given model.

**Table 4:** Age associations with the LDL-C levels in each age phase adjusted for confounding factors.

Female					Male			
Independent variable	Coef.	95% CI	*P*		Independent variable	Coef.	95% CI	*P*
*Age phase I: 18 ≤ age ≤ 56*					*Age phase I: 18 ≤ age ≤ 33*			
Age	0.018	0.018~0.019	<*0.001*		Age	0.025	0.021~0.030	<*0.001*
Systolic BP	/	/	*/*		Systolic BP	/	/	*/*
Diastolic BP	0.004	0.002~0.007	<*0.001*		Diastolic BP	/	/	*/*
Glucose (mmol/L)	0.115	0.105~0.125	<*0.001*		Glucose (mmol/L)	0.169	0.153~0.186	<*0.001*
BMI (kg/m^2^)	0.041	0.032~0.050	<*0.001*		BMI (kg/m^2^)	0.071	0.055~0.087	<*0.001*
Inactive lifestyle	0.043	0.032~0.089	<*0.001*		Inactive lifestyle	0.021	0.011~0.067	<*0.001*
Moderate drinking	/	/	*/*		Moderate drinking	0.031	0.012~0.071	<*0.001*
Smoking	/	/	*/*		Smoking	0.075	0.061~0.079	*0.002*
					*Age phase II: 34 ≤ age ≤ 56*			
					Age	/	/	*/*
					Systolic BP	/	/	*/*
					Diastolic BP	0.007	0.003~0.010	<*0.001*
					Glucose (mmol/L)	0.030	0.022~0.037	<*0.001*
					BMI (kg/m^2^)	0.035	0.023~0.048	<*0.001*
					Inactive lifestyle	0.023	0.013~0.037	*0.004*
					Moderate drinking	0.061	0.054~0.201	*0.003*
					Smoking	0.103	0.108~0.232	*0.001*
*Age phase II: age ≥ 57*					*Age phase III: age ≥ 57*			
Age	-0.013	-0.016~-0.011	*0.001*		Age	-0.008	-0.010~-0.005	<*0.001*
Systolic BP	/	/	*/*		Systolic BP	/	/	*/*
Diastolic BP	0.012	0.007~0.018	<*0.001*		Diastolic BP	0.004	0.001~0.007	*0.002*
Glucose (mmol/L)	0.051	0.019~0.083)	<*0.001*		Glucose (mmol/L)	0.038	0.026~0.050	<*0.001*
BMI (kg/m^2^)	0.024	0.010~0.038	*0.002*		BMI (kg/m^2^)	0.025	0.010~0.040	<*0.001*
Inactive lifestyle	0.021	0.016~0.037	*0.007*		Inactive lifestyle	0.034	0.020~0.043	*0.004*
Moderate drinking	/	/	*/*		Moderate drinking	0.043	0.032~0.077	*0.009*
Smoking	/	/	*/*		Smoking	0.023	0.016~0.052	*0.008*

For abbreviations, see Table 1 and 2. In each multivariate linear regression model, potentially independent variables according to the univariable analysis (p<0.10, see Table 2) were introduced into the starting model and then eliminated manually using the backward step-by-step approach, depending on the largest*P* value. "/" indicates that a variable is not significantly associated with LDL-C level in given model.

### Range-specific correlations between age and LDL-C level with adjustment for confounding factors

The effect of confounding factors on the associations between the LDL-C level and age was examined in multivariate linear regression analyses using the above stated age ranges.

Among females, the LDL-C level increased with age from 18 to 56 years of age with a coefficient of 0.018 and decreased at ≥ 57 years of age with a coefficient of -0.013 (Table 4). Diastolic BP, glucose, BMI, and an inactive lifestyle were independently and positively associated with the LDL-C level irrespective of age (Table 4).

Among males, age was independently associated with the LDL-C level; the association was positive in males ≥ 18 to ≤ 33 years, with a coefficient of 0.025, and negative in males ≥ 57 years of age, with a coefficient of -0.008. Age was not an independent factor in males ≥ 34 to ≤ 56 years old (Table 4, right). Glucose, BMI, an inactive lifestyle, moderate drinking, and smoking were associated with LDL-C level irrespective of age. Diastolic BP was associated with LDL-C level in males ≥ 34 years.

In summary, age remained an independent factor associated with LDL-C levels; the association was positive in males aged 18 to 33 years old and in females aged 18 to 56 years old, and the association was negative in both sexes older than 56 years. Glucose, BMI and an inactive lifestyle were common factors positively associated with LDL-C level irrespective of sex and age.

### Effects of covariate adjustment on the correlation of the non-HDL-C level with age

We next assessed the effect of covariate adjustments on the association between non-HDL-C level and age. Similar to LDL-C, age was independently associated with non-HDL-C levels; the association was positive in females < 57 and in males <34 years old with coefficients of 0.031 and 0.053, respectively, and the association was negative in females and in males ≥ 57 years old with coefficients of -0.015 and -0.018, respectively (Table 5). Glucose level, BMI and an inactive lifestyle were common independent factors associated with non-HDL-C levels for both sexes irrespective of age. Diastolic BP was an independent factor associated with non-HDL-C levels in females regardless of the age range. In contrast, diastolic BP was positively associated with non-HDL-C levels only in males ≥ 34-years old; in males ≥ 18 to ≤ 33 years old, systolic BP was an independent factor positively associated with non-HDL-C levels. Additionally, smoking and alcohol consumption were associated with the non-HDL-C level in males irrespective of age (Table 5).

**Table 5:** Age associations with the non-HDL-C level in each age phase adjusted for confounding factors.

Female					Male			
Independent variable	Coef.	95% CI	*P*		Independent variable	Coef.	95% CI	*P*
*Age phase I: 18 ≤ age ≤ 56*					*Age phase I: 18 ≤ age ≤ 33*			
Age	0.031	0.029~0.032	<*0.001*		Age	0.053	0.049~0.056	<*0.001*
Systolic BP	/	/	*/*		Systolic BP	0.004	0.001~0.008	<*0.001*
Diastolic BP	0.030	0.023~0.039	<*0.001*		Diastolic BP	/	/	*/*
Glucose (mmol/L)	0.191	0.179~0.202	<*0.001*		Glucose (mmol/L)	0.319	0.299~0.339	<*0.001*
BMI (kg/m^2^)	0.056	0.044~0.064	<*0.001*		BMI (kg/m^2^)	0.085	0.067~0.103	<*0.001*
Inactive lifestyle	0.029	0.019~0.045	<*0.001*		Inactive lifestyle	0.034	0.027~0.043	*0.008*
Moderate drinking	/	/	*/*		Moderate drinking	0.037	0.023~0.058	<*0.001*
Smoking	/	/	*/*		Smoking	0.111	0.091~0.175	*0.006*
					*Age phase II: 34 ≤ age ≤ 56*			
					Age	/	/	*/*
					Systolic BP	/	/	*/*
					Diastolic BP	0.008	0.004~0.011	<*0.001*
					Glucose (mmol/L)	0.181	0.172~0.188	<*0.001*
					BMI (kg/m^2^)	0.064	0.049~0.078	<*0.001*
					Inactive lifestyle	0.087	0.081~0.092	<*0.001*
					Moderate drinking	0.031	0.029~0.043	*0.009*
					Smoking	0.118	0.111~0.132	<*0.001*
*Age phase II: age ≥ 57*					*Age phase III: age ≥ 57*			
Age	-0.015	-0.017~-0.012	<*0.001*		Age	-0.018	-0.020~-0.016	<*0.001*
Systolic BP	/	/	*/*		Systolic BP	/	/	*/*
Diastolic BP	0.012	0.006~0.018	<*0.001*		Diastolic BP	0.005	0.001~0.006	*0.004*
Glucose (mmol/L)	0.073	0.036~0.110	<*0.001*		Glucose (mmol/L)	0.117	0.103~0.131	<*0.001*
BMI (kg/m^2^)	0.039	0.023~0.055	<*0.001*		BMI (kg/m^2^)	0.043	0.027~0.060	<*0.001*
Inactive lifestyle	0.028	0.017~0.033	*0.007*		Inactive lifestyle	0.032	0.021~0.068	*0.008*
Moderate drinking	/	/	*/*		Moderate drinking	0.021	0.018~0.034	*0.001*
Smoking	/	/	*/*		Smoking	0.019	0.018~0.034	*0.007*

For abbreviations, see Table 1 and 2. In each multivariate linear regression modeling, potentially independent variables according to the univariable analysis (p<0.10, see Table 3) were introduced into the starting model and then eliminated manually using the backward step-by-step approach, depending on the largest P value. "/" indicates that a variable is not significantly associated with non-HDL-C level in given model.

## DISCUSSION

Few studies have evaluated the age-related changes in lipid levels in a large population with single-year age stratification. Prior studies involved subjects with a narrow age range, stratified the subjects into 5- or 10-year age groups, or performed analyses adjusted for age. Our results showed that the LDL-C/non-HDL-C levels increased from 18 to 56 years of age and decreased thereafter in females, and the LDL-C/non-HDL-C levels increased from 18 to 33 years of age, plateaued at 34 to 56 years of age, and decreased at ≥ 57 years of age in males. Nevertheless, partial consistency could be found with existing reports. In the 1976-1980, 1988-1994, and 1999-2002 National Health and Nutrition Examination Surveys [21], the LDL-C level in males displayed increasing and decreasing trends in those 20 to 29 and 60 to 74 years of age, respectively. The Cardiovascular Health Study also reported a decreasing trend of LDL-C levels in males and a fluctuating decreasing trend in females > 65 years of age [22]. The Very Large Database of Lipids 10B study showed that the median non-HDL-C level increased from the 30 to 39 year old age group to the 40 to 49 year old age group in males, with a subsequent decrease at ≥ 50 years of age; the median LDL-C levels displayed similar trends; among females, the LDL-C level fluctuated with age, while the non-HDL-C level increased from the 30 to 39 year old age group to the 50 to 59 year old age group and subsequently decreased at ≥ 50 years of age [23]. A recent study using quadratic models revealed that, in males, the LDL-C and non-HDL-C levels show nonlinear trends with age irrespective of adjustment for the level of cardiorespiratory fitness [24]. In conclusion, these previous studies failed to comprehensively determine age-related lipid trends; the use of an insufficient sample size and inappropriate age stratification might mask the complete correlation patterns between age and blood lipid levels, while quadratic modeling is not convenient for clinicians to understand the trends of lipid levels with aging.

Based on our results, we propose the following suggestions to improve the current definition, prevention and therapy of dyslipidemia: 1) the definition of optimal, borderline, and high LDL-C and non-HDL-C should be based on age ranges and sex; 2) a lack of awareness of age-related lipid trends might be one possible issue causing uncertainty regarding the role of blood lipid parameters in CHD risk estimators, so current estimators should be refined by age ranges; 3) age range grouping must be implemented in current treatment aimed at lowering plasma LDL-C levels to eliminate the possible effects of aging on blood lipid levels; and 4) a well-grounded age stratification and matching are necessary to ensure the accuracy of observational, interventional and prospective studies focused on lipid-related pathophysiology.

In terms of metabolism, the aging process is characterized by changes in body composition and insulin resistance with declines in the production of growth hormone, insulin-like growth factor-1, and sex steroids [25,26]. These aging-related physiological changes impact lipid metabolism and likely shape the trends of LDL-C and non-HDL-C levels with age revealed in this study, as adjustment for BP, glucose level, BMI, lifestyle, alcohol consumption, and smoking had little effect on the correlation patterns between age and LDL-C and non-HDL-C levels.

The uneven distribution of variables, single time point association and potential reverse causation are three major limitations of observational studies. At the design stage, we tried to avoid these limitations. The large-scale sample size could improve the uneven and nonrandom distribution of variables; age as an independent variable might avoid “reverse causation” between age and lipids. The limitations of this report include the single-center study design; additionally, the contributions of adipose and lean tissue to the variations in lipid and lipoprotein levels could not be distinguished [27] since we adjusted for BMI only; other lifestyle factors, such as eating habits [28,29], were not analyzed; and, as the associations of BP, glucose, BMI and lifestyle with lipid levels have been studied extensively, we omitted this discussion due to space constraints.
